# Shifting the mindset culture to address global educational disparities

**DOI:** 10.1038/s41539-023-00181-y

**Published:** 2023-08-29

**Authors:** Cameron A. Hecht, Mary C. Murphy, Carol S. Dweck, Christopher J. Bryan, Kali H. Trzesniewski, Fortunato N. Medrano, Matt Giani, Pratik Mhatre, David S. Yeager

**Affiliations:** 1 The University of Texas at Austin, Austin, TX USA; 2 Indiana University, Bloomington, IN USA; 3 Stanford University, Palo Alto, CA USA; 4 University of California, Davis, CA USA

**Keywords:** Human behaviour, Education

## Abstract

Educational outcomes remain highly unequal within and across nations. Students’ mindsets—their beliefs about whether intellectual abilities can be developed—have been identified as a potential lever for making adolescents’ academic outcomes more equitable. Recent research, however, suggests that intervention programs aimed at changing students’ mindsets should be supplemented by programs aimed at the changing the mindset *culture*, which is defined as the shared set of beliefs about learning in a school or classroom. This paper reviews the theoretical and empirical origin of the mindset culture and examines its potential to reduce group-based inequalities in education. In particular, experiments have identified two broad ways the mindset culture is communicated by teachers: via informal *messages* about growth (e.g., that all students will be helped to learn and succeed), and formal *opportunities* to improve (e.g., learning-focused grading policies and opportunities to revise and earn credit). New field experiments, applying techniques from behavioral science, have also revealed effective ways to influence teachers’ culture-creating behaviors. This paper describes recent breakthroughs in the U.S. educational context and discusses how lessons from these studies might be applied in future, global collaborations with researchers and practitioners.

Education is a pathway to better health, wealth, and wellbeing^1^, yet educational achievement and attainment remain highly unequal within and across many nations^2^. For example, in the U.S., students of color and students from working-class backgrounds tend to complete less advanced math and science coursework throughout high school^3–8^, and are less likely to earn a college degree^9,10^, compared to peers from racial or ethnic majority groups and from economically advantaged family backgrounds. Similar educational disparities appear globally: for example, a 2015 international assessment found large and persistent socioeconomic disparities in academic achievement in all 72 countries examined^11^.

Over the past several decades, psychological researchers have attended to students’ *fixed mindsets*—the belief that intellectual abilities are fixed—as one factor related to educational inequality^12,13^. Fixed mindset beliefs can come from cultural stereotypes about which groups have high academic potential^14^, and can in turn sustain inequalities by leading minoritized students to believe that they cannot succeed even with great effort. One possible way to promote more equitable outcomes, then, could be to reduce fixed mindset beliefs by encouraging students to adopt a *growth mindset*. This is the belief that students can meaningfully develop their intellectual abilities, under the right conditions (e.g. effort, effective strategies, and support from others)^12,13^. When students endorse (or are encouraged to adopt) more of a growth mindset, they have been found to be more likely to engage in learning-oriented behaviors that lead to improved educational outcomes^12,13^. This has been especially true among students from structurally disadvantaged groups (i.e., those stereotyped by majority groups or excluded from access to high-quality schooling) and students with a history of poorer academic performance^15–19^.

In recent years, there has been growing awareness of the important role that teachers play in supporting students’ growth mindsets^17,20–27^. Teachers are the primary authority figures in the classroom (e.g. they usually set and execute grading schemes), and therefore their practices have a potent impact on the classroom culture^22,23,28–31^. (We define the classroom culture as the shared system of beliefs, goals, and norms that define what it means to be a learner in that classroom^20,23,25,32–34^; also see^35^.) Because research has found that a teacher’s mindset culture is associated with the magnitude of the group disparities in achievement in their classrooms^20^, one of the most important next steps for growth mindset research is to develop and evaluate programs that help teachers improve their mindset cultures.

Accomplishing this goal will require researchers to address several major conceptual and empirical challenges. Here, we detail these challenges and provide a framework for a systematic program of research intended to overcome them, thus paving the way for more equitable student outcomes. We then present recent work that has begun to take this approach and illustrate how it has already yielded breakthroughs in the U.S. educational context, both for students and for teachers. Finally, we highlight the next set of questions that will need to be addressed to broaden this work’s impact beyond the Western contexts that have received the most attention to date.

## Background

### Growth mindsets

Research over the last four decades has indicated that students’ growth mindset beliefs positively predict academic outcomes and can be a fruitful target for interventions^12,13,17,18,36,37^. A growth mindset is a simple but powerful belief because it generates a broader “meaning system” of goals, attributions, and other related beliefs, such as beliefs about the meaning of effort^12,22,38^. For example, students who believe that their intellectual abilities can be developed (growth mindset) tend to seek out challenging opportunities that will build their mastery, even if the learning process involves mistakes or setbacks in the short-term. Students who believe their intellectual abilities are fixed (fixed mindset), however, tend to avoid these opportunities, worried that any such failure would reveal a fundamental lack of competence. Similarly, students in more of a growth mindset tend to attribute failure to controllable factors like strategies and effort, whereas students in more of a fixed mindset tend to see failure as indicating a lack of potential to succeed. Lastly, students who believe that their abilities can grow tend to believe that effort is a tool that will promote such growth, whereas students in more of a fixed mindset tend to believe that the need for effort reveals a fixed lack of ability^12,39^. In turn, the growth mindset meaning system of learning-oriented goals, positive attributions, and positive effort beliefs predicts both short-term academic behavior and downstream academic performance^12,38,40^.

However, growth mindset beliefs (and interventions that promote these beliefs) do not have the same effects for all students in all contexts. Meta-analyses and large national studies indicate that growth mindset effects are heterogeneous, varying meaningfully across students and academic contexts^18,36,37^. In particular, intervention effects have been strongest (a) for structurally disadvantaged students and lower-performing students (suggesting that growth mindset interventions can address inequalities), and (b) in contexts that support and reinforce the intervention message^22,39^. For example, a large nationally representative experiment tested a growth mindset intervention among U.S. 9^th^ graders and found that the intervention improved lower-performing students’ grades, with larger effects on math grades when students’ math teacher also endorsed a growth mindset, but not when the teacher endorsed a fixed mindset^17,18^. These findings suggested that teachers may be a high-leverage target for interventions; that is, for carefully crafted and rigorously tested trainings that help them to create academic contexts that support and reinforce student growth mindset effects^41^.

### From teacher beliefs to classroom cultures

Recent theorizing suggests that it is not simply what teachers privately believe about students’ abilities that can matter for inequality, but what they say and do to create a growth versus fixed mindset culture^22,23,28–31^. For example, teachers who endorse more of a growth mindset tend to use learning-focused practices^17^, such as allowing students to revise and resubmit their work, or explaining that their rigorous standards are rooted in their belief that all students can learn. When students pick up on these cues^42,43^ and perceive their teacher to support a growth mindset, this can influence their experience of psychological safety or vulnerability within the classroom. A recent set of experimental and correlational studies found that students who perceived their teacher to support more of a growth mindset, as compared to a fixed mindset, reported a greater sense of belonging in the teacher’s course, as well as reduced evaluative concerns, reduced feelings of being an “imposter,” and reduced negative affect^21^ (see^44^ for related findings in workplace settings). This may be especially true for students from groups that are negatively stereotyped in terms of intelligence or ability, and who may fear that these stereotypes will inform their fixed mindset teacher’s assumptions about which students are capable^45,46^. A teacher’s growth mindset, on the other hand, implies that *all* students can learn and improve, which may allay concerns about being negatively evaluated and confirming negative stereotypes.

The classroom’s mindset culture, in turn, can affect inequality in two ways. First, there can be a direct link to student achievement disparities. Demonstrating this, researchers at a large four-year university measured the mindset beliefs of 150 professors in the science, technology, engineering, and mathematics (STEM) fields and assessed the link between these teachers’ mindset beliefs and the academic outcomes of their >15,000 students^20^. Instructors’ mindset beliefs—a proxy for the kinds of teacher practices that help build a growth mindset classroom culture—were associated with racial/ethnic achievement disparities. In courses taught by instructors with more of a fixed mindset, underrepresented racial/ethnic minority students received grades that were 0.19 GPA points lower than their racial/ethnic majority peers, on average, whereas this gap was roughly half of that in courses taught by instructors with more of a growth mindset (0.10 GPA points)^20^.

A second way that growth mindset classroom cultures can also address inequalities is by providing “psychological affordances” for students’ own growth mindset beliefs^22,47^. Students’ mindset beliefs have the potential to be an asset in a learning environment, prompting learning-oriented behaviors that can facilitate positive academic outcomes^12,38,40^. When these beliefs are undermined or contradicted within a classroom (i.e., in a fixed mindset classroom culture), they may be less likely to guide students’ behavior within that context and promote learning. However, when growth mindset beliefs are *afforded* (i.e., supported and reinforced) by the environment, students can benefit more from these beliefs. An important consequence of this is that students may profit more from learning the growth mindset (e.g., from an intervention activity) when the school or classroom environment is consistent with the growth mindset message^17,18^.

## Changing classroom cultures with a systematic program of research

How can growth mindset classroom cultures be fostered and maintained? A research agenda that answers this question would have clear relevance for mindset science and practical implications for addressing educational inequalities. In addition, the lessons from such a body of research would also be of wider relevance to behavioral scientists. The effects of many constructs and interventions that are widely studied in behavioral science (e.g., “nudges,” “wise” interventions) vary greatly across individuals and contexts^48–50^. Thus, research on the mindset classroom culture could serve as a model for how to identify and alter aspects of the context that may moderate these effects.

**Fig. 1:**
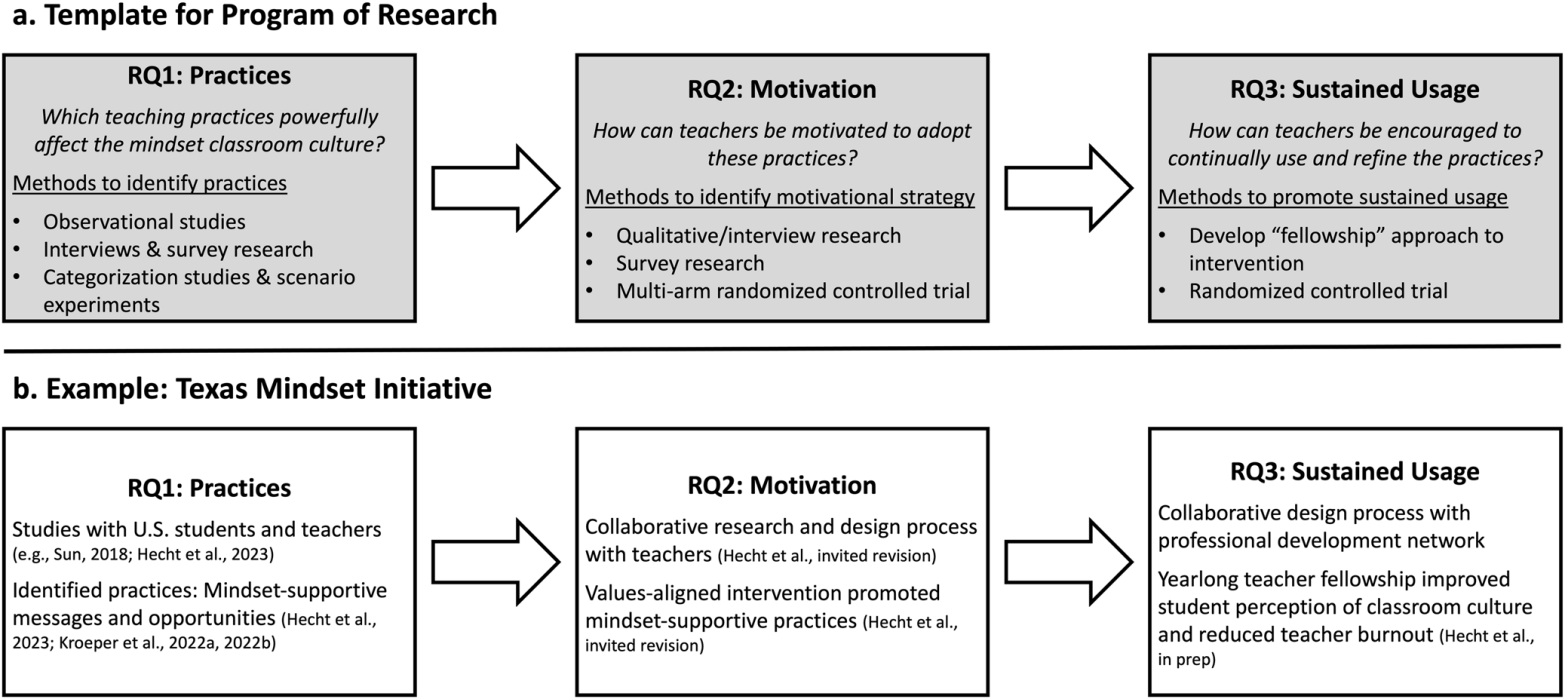
Overview of the research program. General template for a program of research intended to yield an approach to help teachers create and sustain growth mindset classroom cultures (**a**). The approach involves sequentially addressing three research questions (RQs): identifying practices that can/may effectively foster a growth mindset classroom culture (RQ1), finding a way to motivate teachers to adopt these practices (RQ2), and helping teachers to sustain their use of these practices and continue to refine them over time (RQ3). We have piloted this program via the Texas Mindset Initiative (**b**), which developed and evaluated a teacher-focused intervention in Texas public schools.

Here, we propose a template for a program of research (Fig. 1a) that is intended, as its end goal, to yield an effective approach to helping teachers create and sustain growth mindset classroom cultures that narrow achievement disparities experienced by members of structurally disadvantaged groups. This template involves answering three sequential research questions (RQs), discussed below, each of which poses methodological and logistical challenges.

**What are the practices?** The first major challenge to promoting growth mindset classroom cultures is identifying the specific *teaching practices* that can foster such a culture (RQ1). Teaching practices are perhaps the most direct route by which to shift the classroom culture because these practices are directly visible to students and provide important information about the teacher’s beliefs and values^23,42^. Teachers may frequently be called upon to address issues related to the learning process such as the meaning and causes of academic struggle, the meaning and role of mistakes, the meaning of exam or assignment grades, and so on. When teachers’ practices address these issues in a growth mindset-consistent way, they can serve to counter and replace students’ fixed mindset-consistent expectations—expectations that may be especially prevalent among individuals from groups that are stereotyped as having fixed low levels of academic ability^14^. For example, a teacher who explicitly describes struggle as a normal part of the process by which all students can learn and master the material is also contradicting the idea that struggle indicates a fixed lack of ability. Thus, growth mindset-supportive practices may be helpful not only because they promote a growth mindset classroom culture, but also because they help to preclude a fixed mindset classroom culture. Operationally, we define growth mindset-supportive practices as those that (a) clearly and consistently lead students to perceive their teacher as endorsing a growth mindset and (b) provide affordances (i.e., opportunities or support) for students to benefit from their *own* growth mindset beliefs^22^.

**What can motivate behavior change?** The second major challenge, once relevant practices have been identified, is to find effective ways to motivate teachers to adopt these practices (RQ2). Many of the most well-funded attempts to change teachers’ practices (including conventional professional development workshops and evaluation-and-accountability schemes) have failed to produce meaningful effects on student outcomes^51–54^. The reason is not that teachers do not want to improve their classrooms, but instead that they have limited time, face competing priorities, and are exposed to an ongoing “hype cycle” that promotes a rotating array of “new research-based practices”^55^ (see^25^ for a fuller discussion of these barriers).

**How can change be sustained?** Once teachers are motivated to try the suggested practices, the third challenge is to channel this initial motivation into sustained usage and continual refinement of the practices over time. Promoting sustained usage of practices poses a unique challenge, above and beyond fostering an initial motivation, because individuals often return to old routines and habits after initial changes in behavior^56^. Indeed, “fadeout” of a variety of educational and psychological interventions is well documented, sometimes even when these interventions bring initial success^57–59^.

## An illustrative case: the Texas mindset initiative

Here, we illustrate how the three research questions and related challenges noted above can be addressed, drawing on a series of studies conducted by the Texas Mindset Initiative (TxMI), as well as other related studies (see Fig. 1b). TxMI is a collaborative, interdisciplinary endeavor undertaken by researchers housed in several universities across the U.S. (including the authors of the present paper), with the goal of developing an effective approach to promoting growth mindset classroom cultures in the state of Texas. Texas is a good initial site in which to pursue this research agenda because (a) it is reasonably representative of the U.S. in terms of racial/ethnic minority groups and income disparities, (b) its state government has been resistant to direct efforts to promote diversity and is therefore an important context to evaluate whether promoting growth mindset classroom cultures can help to address group-based inequalities, and (c) Texas has unique state student data systems that make it easier to track students longitudinally with minimal nonresponse. The studies reviewed here highlight breakthroughs in the goal of shifting classroom cultures while also providing an example of how to address similar research questions related to intervening with teachers.

### RQ1: Identifying growth mindset-supportive practices

The first challenge in identifying growth mindset-supportive teaching practices was to identify candidate practices among those that are used by teachers. Sun (2018) collected videotaped class sessions with middle school math teachers who had reported relatively high levels of fixed or growth mindset beliefs^31^. The researcher identified four categories of practices that characterized these teachers: (1) practices that inform how students are classified and compared (e.g., expressing high expectations for all students vs. only some students^60^), (2) practices that set norms related to learning and improvement (e.g., treating mistakes as learning opportunities rather than indicators of low ability^61^), (3) practices related to engagement with the material (e.g., student driving the work with teacher support vs. teacher simply demonstrating^62^), and (4) practices related to feedback and assessment (e.g., praising process rather than ability^63^).

Following up on these observational findings, TxMI researchers identified expert high-school teachers from within a large professional development network. The goal was to find growth mindset practices that already seem to be working in real classrooms, to avoid the possibility of introducing practices that are only rooted in theory but do not have a chance of working in the complexity of everyday teaching. Our team estimated a model predicting the “value added” of teachers in a sample of over 18,000 students, which refers to the positive residual score for student achievement after accounting for student prior achievement and demographics (i.e., race/ethnicity, gender, and parental education). Teachers who vastly overperformed the model’s predictions were labeled “bright spots” and they were were invited to participate in a workshop to help us learn about how they promoted growth mindset-supportive learning environments. A qualitative analysis of the workshop revealed a set of practices that was consistent with the observational findings from Sun^31^ while providing further insight into these teachers’ perspectives and strategies for implementing these practices effectively (to be reported in a future paper). Together, the observational research and “bright spots” approaches provided an initial set of candidate practices that were common in the classroom, as well as clear directional hypotheses regarding which type of mindset they might communicate. However, this work left open the question of which practices students *perceive* to communicate information about a classroom’s mindset culture.

Kroeper and colleagues conducted a series of qualitative, survey, and experimental studies to examine whether students perceived particular teacher practices to clearly communicate a growth or fixed mindset^29,30^. The authors conducted focus groups in which college students reported practices and behaviors that characterized their previous teachers whom they had perceived to hold a fixed or growth mindset. Students referenced many of the practices identified in the previous observational research. The researchers then confirmed the findings from these focus groups across two studies. First, they conducted a survey in which college students reported their perceptions of an instructor’s mindset beliefs and how often their instructor used the practices identified in the prior focus groups^30^. Second, the researchers asked college students to categorize 119 specific teaching practices as indicating a growth or fixed instructor mindset^29^.

In both studies, the identified practices were associated with students’ perceptions of the instructor’s mindset beliefs in the expected directions, and the categorization study provided further insight into which practices were most consistently associated with these perceptions. For example, expressing a belief that anyone could be a top student in the class, discussing ways for students to improve their grades over the course of the semester, and encouraging students to ask questions to improve their understanding were all categorized as indicating a growth mindset by ≥99% of students. Conversely, expressing a belief that only some students could succeed, encouraging struggling students to drop the course, and discouraging questions during class time were categorized as indicating a fixed mindset by ≥99% of students.

Building on this foundation, TxMI researchers developed a laboratory paradigm to test the causal effects of a hypothetical teacher’s practices. Across four studies, Hecht and colleagues^28^ manipulated descriptions of teachers’ practices to be supportive of a growth or fixed mindset (using a selection of those identified in the research reviewed above; see Table 1). The authors’ manipulation included two broad categories of practices that each uniquely contributed to the perceived mindset culture: teachers’ verbal messages (i.e., what they say that supports a growth or fixed mindset) and the opportunities they provide for students to enact a growth mindset (i.e., policies that emphasize learning, such as the opportunity to revise and resubmit assignments for credit; see^28^ for related research on supportive classroom structures).

**Table 1:** Teacher statements in experiment manipulating classroom mindset culture.

Experimental Condition	Messages	Opportunities
Growth mindset teacher-led classroom culture	E.g., “This class is set up the way it is because I believe that all students can learn and do well in the class, no matter where they started out.”	E.g., “If you show improvement in your exam grades over the course of the term, I’ll raise your final grade.”
Fixed mindset teacher-led classroom culture	E.g., “Students who do the best at the beginning of the year are typically the same ones who do well at the end.”	E.g., “You cannot make up for problems missed on previous tests, so make sure you’re prepared for each test.”

Experimental stimuli from scenario studies by Hecht and colleagues^28^.

After presenting adolescent participants with manipulations of these practices, the authors measured (a) students’ perceptions of the classroom culture and (b) students’ willingness to engage in learning-oriented behaviors that tend to be associated with students’ growth mindset beliefs. Participants perceived the intended differences in the growth mindset classroom culture (based on the manipulated teacher practices). Critically, the teacher practices manipulation also affected participants’ willingness to act upon their own growth mindset beliefs. Whereas there was a positive association between participants’ growth mindset beliefs and learning-oriented choices in the growth mindset teacher’s class (e.g., choosing more challenging coursework that would promote learning), this association was nullified in the fixed mindset teacher’s class. Students did not opt to put their growth mindset into practice in that classroom. Importantly, the effects were strongest when teachers provided *both* growth mindset-supportive messages and opportunities, rather than one or the other (see Study 3)^28^. Interestingly, the effects of the teacher practices manipulation did not depend on students’ race/ethnicity, socioeconomic status, or gender (see Study 2), suggesting that clear, unambiguous growth mindset-supportive cues may be perceived similarly across groups of students.

In sum, observational, interview, survey, and experimental research enabled the TxMI team to systematically winnow down the broad pool of practices that instructors in the U.S. use and to pinpoint those practices that most potently affect the growth mindset classroom culture, to categorize these practices into two simple and useful dimensions, and to establish a causal link between these practices and students’ experiences and choices.

### RQ2: Motivating teachers to adopt growth mindset-supportive practices

As described earlier, many aspects of the teaching profession make behavior change especially difficult to promote^25^. To address this challenge, TxMI researchers drew on a framework known as “values alignment,” which has proven effective at changing behavior in domains where most other approaches have failed (e.g., encouraging adolescents to choose healthier foods^64,65^). Values alignment reframes a given behavior in terms of how it serves a core value that a person already holds. People’s core values are often defined, at least in part, by shared standards within a social group (e.g., among teachers) for what constitutes a person who is worthy of respect and admiration in their group or setting^66^. By connecting a behavior with these shared standards, values alignment imbues the behavior with a sense of motivational priority.

**Fig. 2:**
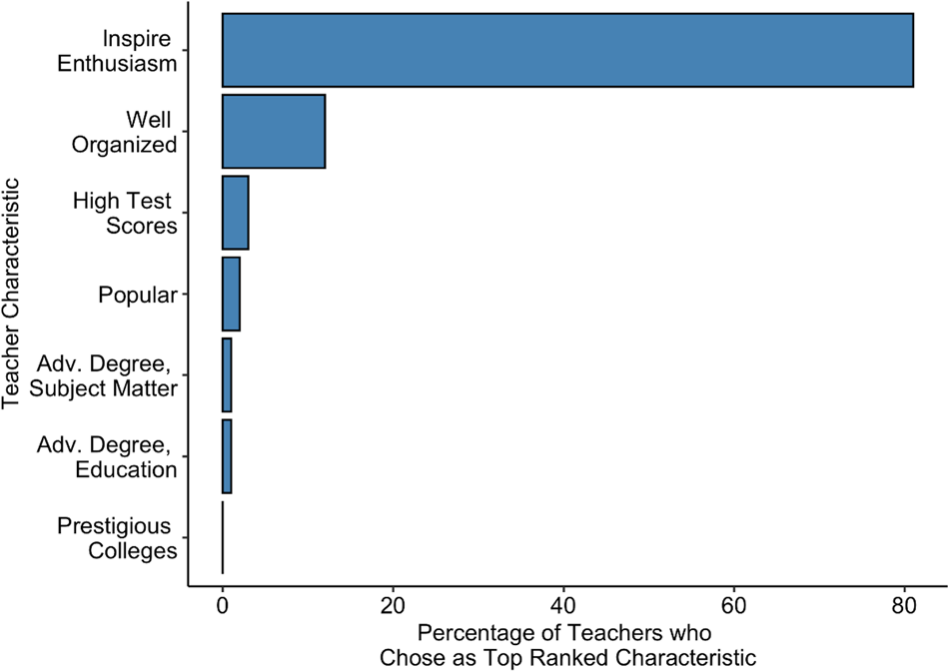
Teachers’ endorsement of professional values. Percentage of teachers who ranked each of seven possible characteristics as the most important for earning the professional respect of their colleagues (adapted from^67^). Inspiring students’ enthusiasm for learning was ranked as the most important characteristic by 81% of teachers and in the top 3 by 97% of teachers. Other characteristics included being well organized, having high average student standardized test scores, being well liked by students, having an advanced degree in their subject matter, having an advanced degree in education and teaching, and frequently helping their most promising students get into prestigious colleges.

Hecht and colleagues^67^ used the values-alignment framework to develop a growth mindset-supportive teaching intervention, informed by initial qualitative work, followed by confirmatory survey research. The authors conducted a series of in-depth focus groups with high school teachers across the state of Texas with the goal of identifying a core value that these teachers shared. Through these interviews, the authors found a widely shared, deeply held value: being able to inspire their students’ enthusiasm for learning without needing to coerce them with threats or yelling. To confirm this initial conclusion, the researchers conducted a survey with a nationally representative sample of 965 high school math instructors in which they were asked to rank each of seven characteristics in terms of the degree to which it helped a teacher to earn the professional respect of their colleagues. The characteristics included the value identified during the interview phase, as well as six other distractor values (e.g., having high average standardized test scores). The results unambiguously confirmed that inspiring students’ enthusiasm for learning was a core value among teachers: 81% of teachers in the sample ranked this as the most important characteristic and 97% of teachers ranked it within the top three (Fig. 2)^67^.

The researchers next developed a ~45-min, self-administered, online values-aligned treatment module for teachers. The first half of the intervention used a values-aligned argument that connected growth mindset-supportive practices with the identified core value of inspiring students’ enthusiasm for learning. The second half of the intervention provided guidance on exactly how teachers could use the suggested growth mindset-supportive practices. Throughout the intervention development process, the researchers consulted with several of the “bright spot” teachers (described above) to refine the intervention argument and ensure that it would resonate with other high-school teachers.

The logic of the values-aligned argument (in the first half of the intervention) was as follows. (1) Hormonal changes in adolescence make teenage students hyper-attuned to cues of respect and disrespect from adults^68–70^. (2) Subtle (often unintentional) cues from teachers can lead students to believe that their teacher does not believe in their potential to learn and improve. (3) Students’ perception that their teacher does not see them as capable of learning can cause them to feel that their teacher sees them as a poor investment of time and energy. (4) This perception leads students to feel disrespected by their teachers. (5) When students feel disrespected by a teacher, they tend to react by either reciprocating the perceived disrespect (e.g., acting out in class) or disengaging from the teacher-student relationship. (6) On the other hand, students’ hyper-sensitivity to cues of respect presents teachers with an opportunity. If teachers provide clear cues of respect—specifically, clearly, and consistently expressing a belief in their students’ potential to learn and improve—students tend to reciprocate this perceived respect by enthusiastically engaging in the class.

In the second half of the values-aligned intervention, the module provided teachers with examples of how teachers can communicate their belief in all students’ potential to learn and improve. The examples were consistent with core tenets of growth mindset theory^71^ and directly informed by the practices identified in RQ1 (above). The intervention used tools that are typical of “wise interventions” that help to promote internalization of the intervention message^58,72^. For example, teachers were given the opportunity to develop growth mindset-supportive messages in their own words^73^ and make concrete plans about how and when to communicate these messages^74^.

Once developed, the values-aligned intervention was tested as part of a multi-arm randomized controlled trial with 897 high-school teachers and their 32,172 students. These teachers were teaching dual-enrollment courses in which students learned college-level content (e.g., College Algebra, Introductory Biology I, Introduction to Rhetoric) and could simultaneously earn high-school and college credit. The teachers were randomly assigned to one of six conditions: a condition in which they received the values-aligned growth mindset-supportive teaching intervention (described earlier), a control condition in which they received training on a topic unrelated to growth mindset, which did not include values-alignment, or one of four other treatment arms that mostly used a pragmatic, informational approach (i.e., not values-aligned) to promote growth mindset-supportive practices. Compared to the control condition and each of the other treatment arms, the values-aligned growth mindset-supportive teaching intervention was more successful in promoting teachers’ motivation to adopt the suggested growth mindset-supportive practices. In addition, this one-session intervention improved students’ end-of-year performance in the course, with stronger effects in classes with a higher proportion of students from lower-SES backgrounds. Specifically, the single-session intervention increased pass rates by four percentage points overall, and by 6 to 11 percentage points in lower-SES classrooms (depending on the model)^67^.

To summarize, through a systematic process of qualitative research and confirmatory survey research in a national sample, TxMI researchers developed a successful intervention strategy to motivate teachers to adopt the growth mindset-supportive practices identified in the prior research, and this intervention helped to reduce socioeconomic inequality in students’ academic outcomes.

### RQ3: Encouraging teachers to continually use and refine the practices

After instilling an initial motivation in teachers to adopt the suggested growth mindset-supportive practices, what supports can be added to help them to continue to use these practices and refine them within their unique classroom environment? To address this question, TxMI researchers developed a “fellowship” program for teachers that would enable multiple points of contact and opportunities for further training throughout the schoolyear (to be reported in a future paper; see^75^ for a related approach that provided extensive training to teachers through a summer institute).

The fellowship was designed to begin with the values-aligned growth mindset-supportive teaching intervention (given before the start of the schoolyear), immediately followed by training sessions (over three days) in which the messages from the intervention were elaborated upon and reinforced. During this time, teachers were also given multiple opportunities to workshop their use of several suggested practices. For example, in small groups, teachers practiced delivering short speeches, intended to be delivered to their students on the first day of class, that emphasized the teacher’s belief that all students could reach the teacher’s high standards, focused on growth and improvement, and made clear the ways in which the teacher would support students in their efforts to improve. As another example, teachers were asked to look for opportunities within their syllabus to build in opportunities for students to be rewarded for their efforts to learn, such as allowing students to revise and resubmit assignments or to correct their mistakes on exams for credit. In this way, these initial sessions helped teachers prepare to provide *both* messages and opportunities that would help to build a growth mindset classroom culture.

Throughout the school year, teachers were provided multiple supports to help sustain changes to their teaching practice. These supports were fully virtual and minimized unnecessary burden that would compromise the scalability of the approach. Teachers had monthly facilitated virtual check-ins with other teacher fellows, as well as virtual full-day Professional Learning Institutes once each semester (fall and spring) to discuss their progress integrating the practices into their classrooms and to provide feedback and suggestions to one another. These sessions helped to provide teachers with a consistent community to support their efforts at transforming their classroom culture, while also serving as a practical resource for their own professional development. In addition, teachers were provided access to an online “library of practices,” that summarized and provided examples of the practices they had been introduced to and encouraged to adopt. They were also encouraged to submit their own ideas for practices to the library when these practices were not already included. In this way, the teachers were given access to a rich source of materials to help them accomplish their goals in the classroom, while also invited to participate as collaborators in the community of teacher fellows (rather than positioning them merely as beneficiaries of the fellowship).

The researchers tested the teacher fellowship in a small-scale pilot (*N* = 57 Algebra I teachers, 3,234 students) during the COVID-19 pandemic. Teachers were randomly assigned to the fellowship or a control group that received a different professional development related to cognitive science. Preliminary analyses indicate that, as compared to teachers in the control group, those who received the fellowship had classroom cultures that were significantly more consistent with and supportive of a growth mindset, as assessed by their students’ ratings on a classroom climate survey (e.g., “In my math class, students are rewarded for trying hard and improving”). In addition, the fellowship seemed to have promising unintended effects for teachers, related to their motivation and persistence. In a year where teacher attrition was at a peak, fewer teachers in the fellowship condition reported seriously considering leaving their job as an Algebra I teacher (12.5%) than those in the control condition (23.1%). Currently, the TxMI team is preparing to scale the fellowship to test the program with a larger sample of high school teachers (*N* = ~ 1000).

In sum, by building a consistent set of structural and communal supports around a strong motivational strategy (the values-aligned growth mindset-supportive teaching intervention), the “fellowship” intervention approach was able to produce changes in teachers’ classroom cultures. In addition, the fellowship seemed to reduce the incidence of teacher burnout, perhaps helping them to shape classrooms that indeed had more eager and motivated students (as suggested in the values-aligned intervention module), making their classrooms into more professionally fulfilling and enjoyable places to work.

## Four pressing questions for the next phase of research

The research reviewed above demonstrates that the goal of transforming classroom cultures is challenging and requires programmatic studies that incrementally answer basic underlying questions. However, there are still important questions that, if answered, could dramatically increase the scale and impact of this work. Here, we describe four of the most pressing issues and preview how the next phase of research might begin to address them.

### Testing the separate and joint roles of student- and teacher-focused interventions

An important step for future research will be to identify the distinct and joint contributions of teachers’ growth mindset-supportive practices and researcher-designed growth mindset intervention activities for students. Changing individuals’ beliefs is a nuanced and challenging undertaking^58,72^. Growth mindset interventions, which use tools informed by social-psychological theories of motivation and persuasion, can consistently instill a growth mindset in students^18,19^, whereas teachers’ attempts to do so may vary in effectiveness. Teachers, however, may be especially well suited to create a psychologically affording classroom culture where these growth mindset beliefs ring true, as illustrated by the research described thus far. Therefore, we suspect that the most effective treatments will combine student-targeted growth mindset interventions and values-aligned trainings that motivate teachers to create growth mindset-supportive classroom contexts. However, this possibility remains untested and important questions remain.

For example, although teachers may struggle to change students’ beliefs with explicit messages or lessons about growth mindset in the short-term, perhaps creating a growth mindset-supportive classroom culture can instill growth mindset beliefs in students over time. If this is the case, it is unclear whether student mindset interventions would meaningfully add to the effects of the type of teacher-focused intervention described here, or if such student interventions would be rendered unnecessary. The existing research cannot tease these possibilities apart. For instance, in the values-aligned teacher intervention study described above, all students in the study received a brief “low-dosage” version of the growth mindset intervention. In future studies, researchers should test the individual and combined effects of the teacher and full-dose student interventions in a crossed experimental design to estimate the influence of each in promoting equitable student outcomes.

### Improving measurement

**Fig. 3:**
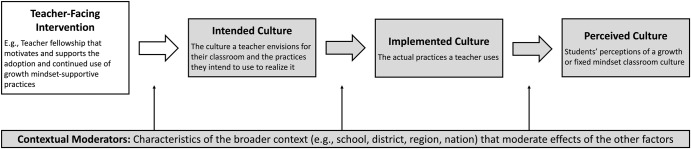
Teacher intervention process model. Process by which a teacher-facing intervention may alter the classroom culture (adapted from^26^). Gray boxes indicate the aspects of educational contexts that must be carefully measured to understand how such an intervention might change the classroom culture.

One important step for future work is to develop more detailed and nuanced measures. There are at least four aspects of educational contexts that must be carefully measured to understand how teacher practices might alter the classroom culture (see Fig. 3)^26^. Briefly, these areas are (a) the intended culture (i.e., the culture a teacher envisions for their classroom and the practices they intend to use to realize it), (b) the implemented culture (i.e., the actual practices a teacher uses), (c) the perceived culture (i.e., students’ perceptions of a growth or fixed mindset classroom culture), and (d) contextual moderators (i.e., characteristics of the broader context, such as the school or district, that moderate effects of the other factors).

The studies reviewed above have found promising effects on measures of the intended and perceived classroom culture. However, the remaining two areas of measurement need improvement in future research. First, validated growth-mindset-specific measures of the implemented culture—what teachers actually do—will be essential to fully understand the processes by which teachers change their classroom cultures. Second, precise measures of contextual moderators of teacher-facing interventions, such as school or district policies that encourage teachers to experiment with new practices (vs. mandate a standardized curriculum or grading scheme) will be needed to understand where and how teachers can most effectively alter their classroom cultures. The TxMI team is currently addressing both of these gaps, exploring avenues by which to obtain and code observational classroom data and developing school- and district-level measures to complement the upcoming scaled-up teacher fellowship trial.

### Developing sophisticated tools to support teacher change

Another area with great potential for growth is developing new, sophisticated tools to assist teachers in their efforts to foster a growth mindset classroom culture. Such tools would likely be more effective to the extent that they are personalized to individual teachers’ needs and while personalized approaches such as one-on-one teacher coaching have been effective^76^, these methods have not been easily scalable. However, the rapid improvement of natural language processing algorithms may soon bring scalable methods into reach^77,78^. Before these tools can be used to assist teachers directly, they must be able to detect and produce growth mindset-supportive language. In a recent study by Clapper and colleagues (to be reported in a future paper), when trained on language produced from effective growth mindset-supportive teachers, these algorithms were able to produce text that adolescents rated similarly to the real teachers (i.e., those who provided the training language) in terms of communicating messages that are consistent with a growth mindset classroom culture.

The most important next step in this research will be to refine these tools so that they can provide feedback to teachers to help make their language more supportive of a growth mindset as they compose messages for their class. Similarly, perhaps this technology could be used to assess transcripts of audio recordings from teachers’ lessons and provide suggested changes for future lessons. The many possibilities for how technology may help teachers to build growth mindset classroom cultures through interactions with their students are exciting.

As these possibilities are realized, it will also be important to systematically anticipate and investigate intended and unintended consequences of these tools (see^79^ for an example of such a systematic framework). For example, this work should explore whether some teachers may come to rely too heavily on the surface-level features of these tools, rather than internalizing a deeper understanding of how to support the growth mindset in the classroom. With a systematic approach that ensures careful and ethical development of such technological supports, these possibilities may eventually be realized in the form of tools that could come to be seen as indispensable for teachers.

### Adapting the approach to other cultural contexts

A recent international study found that students’ growth mindset beliefs were positively associated with academic achievement in 72 of 74 nations that administered the growth mindset survey, with stronger associations among students from structurally disadvantaged backgrounds such as those from lower-SES and immigrant families^39,80^. Therefore, growth mindset support is likely to be a worthwhile target for intervening to address group-based disparities beyond the U.S. and other Western countries. However, educational contexts around the world are likely to differ in their norms, values, available resources, and barriers to change, making adaptation and customization an essential process. Ongoing research by Medrano and colleagues (to be reported in a future paper) is examining how insights from the TxMI research described above can be adapted for teachers in Bangladesh. At the outset of this work, researchers conducted a series of focus groups with Bangladeshi teachers and students, as well as a large-scale survey with 200 teachers and 1000 of their students from 40 schools across three districts. The majority of surveyed teachers (82%) believed that some students have the ability to excel while others do not, suggesting that the mindset classroom culture might indeed be a fruitful target for intervention.

This work revealed key similarities and differences between the U.S. and Bangladeshi contexts. As in the U.S., Bangladeshi teachers expressed a great deal of concern about student engagement. However, this concern seemed to take different forms in the two contexts. Whereas U.S. teachers expressed valuing cultivating high levels of engagement (i.e., inspiring enthusiasm for learning), Bangladeshi teachers expressed concern with low levels of engagement from students collectively known as “back benchers.” For these students, the teachers attributed the lack of progress to external factors such as low parental involvement. The teachers also worried that they lacked the time and resources that would be necessary to help students overcome these barriers (see^81^ for similar findings with teachers across nine developing countries). As a result, the surveyed teachers ranked “inspiring enthusiasm” (the most highly ranked value among U.S. teachers) as their *least* important value, and instead tended to rank students being prepared for class, achieving high average test scores, and being punctual as their highest values.

Whereas U.S. teachers may have the luxury of focusing on fostering high levels of engagement (as students’ most basic educational needs tend to be already met), Bangladeshi teachers are confronted with students who are disengaged due to factors that are outside of the teacher’s control. Thus, although Bangladeshi teachers may want to help “back bencher” students, they may feel helpless to do so, leading them to instead focus on the higher performing students who seem more within reach.

Developing culturally adapted growth mindset-supportive teacher trainings is an exciting new frontier for growth mindset research. Ensuring that these trainings resonate with teachers and are effective for students will require that researchers become intimately familiar with the barriers, constraints, values, and norms of the local context. The TxMI team has begun to lay some of this groundwork in Bangladesh. With additional qualitative and survey research to form and test hypotheses about the context, this work may eventually yield an effective treatment for Bangladeshi teachers as well as a template for how to translate this work to new cultural contexts.

## Transforming classroom cultures

We know that teachers are a powerful lever for addressing inequality in educational outcomes because of their potential to shape the culture of the learning environment. Classrooms characterized by the belief that all students can learn and improve their abilities (i.e., those with growth mindset cultures) are less psychologically threatening and have smaller group-based academic disparities than classrooms characterized by the belief that only some students have the requisite “natural talent” to succeed (i.e., those with fixed mindset cultures)^20,21^. However, we have not known exactly how to move this lever. Changing the classroom culture is challenging and teachers receive minimal formal support to help them conceptualize and realize such a transformation.

The research reviewed here indicates that developing such supports—for example, psychological interventions that are delivered in the form of teacher training—is a major undertaking that requires a systematic agenda and a variety of methods to address different goals along the way. We hope that this series of studies will serve as a useful template for other researchers who are interested in understanding other aspects of the classroom culture and identifying strategic ways to help teachers change the relevant classroom characteristics. We have already made great strides toward realizing this goal in the area of creating growth mindset-supportive learning environments, and the next phase of research promises to build on this progress. Doing so will open the door to developing high-quality and personalized supports that may help a global community of teachers to reduce educational inequalities.

## Data Availability

This paper does not report on any primary data. No dataset is needed to interpret, replicate, or build upon the content of this article.
